# MiRNA Expression Profile of Human Subcutaneous Adipose and during Adipocyte Differentiation

**DOI:** 10.1371/journal.pone.0009022

**Published:** 2010-02-02

**Authors:** Francisco J. Ortega, José M. Moreno-Navarrete, Gerard Pardo, Monica Sabater, Manuela Hummel, Anna Ferrer, Jose I. Rodriguez-Hermosa, Bartomeu Ruiz, Wifredo Ricart, Belen Peral, José M. Fernández-Real

**Affiliations:** 1 Service of Diabetes, Endocrinology and Nutrition, Institut d'Investigació Biomédica de Girona (IdIBGi), CIBEROBN (CB06/03/0010) and Instituto de Salud Carlos III (ISCIII), Girona, Spain; 2 Microarray Unit, Center for Genomic Regulation (CRG), Universitat Pompeu Fabra (UPF), Barcelona, Spain; 3 Instituto de Investigaciones Biomédicas ‘Alberto Sols’ (IIB), Consejo Superior de Investigaciones Científicas (CSIC) and Universidad Autónoma de Madrid (UAM), Madrid, Spain; 4 Department of Surgery, Institut d'Investigació Biomédica de Girona (IdIBGi), Girona, Spain; Hong Kong University, Hong Kong

## Abstract

**Background:**

Potential regulators of adipogenesis include microRNAs (miRNAs), small non-coding RNAs that have been recently shown related to adiposity and differentially expressed in fat depots. However, to date no study is available, to our knowledge, regarding miRNAs expression profile during human adipogenesis. Thereby, the aim of this study was to investigate whether miRNA pattern in human fat cells and subcutaneous adipose tissue is associated to obesity and co-morbidities and whether miRNA expression profile in adipocytes is linked to adipogenesis.

**Methodology/Principal Findings:**

We performed a global miRNA expression microarray of 723 human and 76 viral mature miRNAs in human adipocytes during differentiation and in subcutaneous fat samples from non-obese (n = 6) and obese with (n = 9) and without (n = 13) Type-2 Diabetes Mellitus (DM-2) women. Changes in adipogenesis-related miRNAs were then validated by RT-PCR. Fifty of 799 miRNAs (6.2%) significantly differed between fat cells from lean and obese subjects. Seventy miRNAs (8.8%) were highly and significantly up or down-regulated in mature adipocytes as compared to pre-adipocytes. Otherwise, 17 of these 799 miRNAs (2.1%) were correlated with anthropometrical (BMI) and/or metabolic (fasting glucose and/or triglycerides) parameters. We identified 11 miRNAs (1.4%) significantly deregulated in subcutaneous fat from obese subjects with and without DM-2. Interestingly, most of these changes were associated with miRNAs also significantly deregulated during adipocyte differentiation.

**Conclusions/Significance:**

The remarkable inverse miRNA profile revealed for human pre-adipocytes and mature adipocytes hints at a closely crosstalk between miRNAs and adipogenesis. Such candidates may represent biomarkers and therapeutic targets for obesity and obesity-related complications.

## Introduction

The development of adiposity involves extensive adipose tissue remodeling at the cellular level and is dependent on the coordinated interplay between adipocyte hypertrophy (increase in cell size) and adipocyte hyperplasia (increase in cell number) [**1**]–[4****]. Hyperplasic component of adipose tissue is currently well recognized [**5**]–[**8**] and refers both the recruitment and proliferation of adipocyte precursor cells (also named pre-adipocytes) followed by the complex process by which new fat cells are developed from pre-adipocytes [**1**], [2], [**9**]. Looking for an in-depth understanding of these processes, much attention has been given to the regulators that modulate pre-adipocytes proliferation and differentiation (also named adipogenesis). Both processes are tightly controlled, in negative or positive ways, by a combination of multiple transcription factors [**10**]. Potential regulators of adipogenesis include microRNAs (miRNAs), small non-coding RNAs which represents an abundant class of ∼22-nucleotide length RNAs evolutionarily highly conserved. MiRNAs expression levels have been recently shown as differentially regulated in mice during *in vitro* as well as *in vivo* adipogenesis [**11**].

Since its early discovery in 1993, miRNA expression profiles and functions have been extensively studied. It is believed that miRNAs control the post-transcriptional regulation of ∼30% of mammalian genes [**12**], either via degradation of target mRNAs or by translational repression [**13**]–[**15**]. Through modifying mRNAs availability and protein synthesis, miRNAs control many cellular processes such as cell differentiation [**16**]–[**18**], growth, proliferation and apoptosis [**19**]–[**21**]. Indeed, since miRNAs expression is deeply related to cellular behavior and, eventually, the correct development and function of body tissues, changes in miRNAs expression profiles are being extensively studied in human diseases such as cancer, osteoporosis, cardiac illness and heart failure [**22**]–[**25**]. Moreover, miRNAs are attractive candidates for regulating cell fate decisions and complex diseases since the simultaneous coordination of a large number of target genes, potentially accomplished by a single miRNA, may be a key-factor to defining specific differentiated or pathogenic cell states.

Unlike cancer, few studies have aimed to define a miRNA expression profile for adipose tissue and fat cells [**11**], [26]–[**28**]. Xie et al. [**11**] investigated the regulation and involvement of 373 miRNAs in fat cell development, enlarged fat depots and obesity working with fat samples from mice and *in vitro* cultured 3T3-L1 cells. Otherwise, Klöting et al. [**26**] performed a global miRNA expression study of 155 miRNAs in both subcutaneous and omental human fat depots of overweight and obese individuals. Previously, Kajimoto et al. [**28**] (102 miRNAs) and Esau et al. [**27**] (254 miRNAs) tried to identify by anti-sense inhibition of miRNAs significantly deregulated in fully differentiated adipocytes, key-miRNAs for adipocyte differentiation, working with 3T3-L1 and human fat cells, respectively.

In this study, we have profiled the expression of 799 mature miRNAs (723 human and 76 viral) during adipogenesis of human adipocytes and in subcutaneous adipose tissue samples from lean and obese with and without Type-2 Diabetes Mellitus subjects. We aimed to investigate whether miRNA expression profile in human adipocytes changes during adipogenesis and whether this pattern significantly differs between cells from obese or lean subjects, as well as in subcutaneous fat depots. We found that the expression of 70 miRNAs was modulated in fully differentiated human adipocytes. Fifty miRNAs significantly differed between either pre- or mature adipocytes from lean and obese individuals. We then examined whether modulation of the expression of these miRNAs is also detected in subcutaneous fat depots.

## Methods

### Cell Culture

Cryopreserved human subcutaneous pre-adipocytes (*Zen-Bio®, Inc.*) from both lean (BMI<25.0 Kg/m^2^) and obese (BMI>30.0 Kg/m^2^) subjects were plated, cultured and differentiated according to manufacturers' guidelines as described as Supplemental Information (SI) in *Materials and Methods S1*. Three biological replicates (n = 3 replicates/cell/day) were performed during differentiation (days 0, 7 and 14 after starting differentiation protocol) for each cell line.

### Subjects and Samples

Twenty-eight adipose tissue biopsies were obtained from subcutaneous fat depots of a group of women (n = 28) with Body Mass Indexes (BMI) between 20 and 55 Kg/m^2^. They were invited to participate at the Endocrinology Service of the *Hospital Universitari Dr. Josep Trueta de Girona* (Girona, Spain). Informed written consent was obtained after the purpose, nature, and potential risks of the study were explained to all of them. The experimental protocol was approved by the Ethics Committee of the *Hospital Dr. Josep Trueta of Girona*. Human patient characterization as well as sample preparation is described in *Table S1* and *Materials and Methods S1* files, respectively.

### Microarray and Data Analysis

The analysis of 723 human and 76 viral mature miRNAs (799 miRNAs) was carried out for both *in vitro* and *in vivo* samples. Total RNA, including mRNAs, small RNAs and microRNAs, was extracted, purified and prepared from adipose tissue fragments and cell debris using miRNeasy® Mini Kit *(QIAgen)*. The integrity of each RNA sample was checked with an Agilent Bioanalyzer® *(Agilent Technologies)*. Human miRNA microarrays *(Agilent Technologies)*, containing 13,737 probes corresponding to 799 miRNAs, and 22 control probes, were then hybridized following manufactures' protocol. Microarray preparation, as well as statistical analysis, is described in detail in *Materials and Methods S1*. Within the miRNAs with significant test results we report only those which have normalized (non-logged) signal intensities above 150 in at least half of the samples involved in the respective comparison, and which hence can be trusted not to represent “*noise*”. Complete miRNA microarray data from cells and human fat depots are deposited into Gene Expression Omnibus (GEO) following MIAME compliant guidelines (Accession numbers: GSE18469 and GSE18470, respectively; NCBI tracking system #15713167).

### Gene Expression Analyses

Three µg of total RNA were reverse transcribed to cDNA using High Capacity cDNA® Archive Kit *(Applied Biosystems)* according to the manufacturers' guidelines. MiRNAs were reverse transcribed by TaqMan® MicroRNA Reverse Transcription Kit *(Applied Biosystems)*. Gene expression was assessed by Real Time-PCR using an ABI Prism® 7000 Sequence Detection System and TaqMan® technology suitable for relative gene expression quantification as described in *Materials and Methods S1*.

Cyclophilin A (*PPIA*) and RNU48 were used such as endogenous controls in each reaction for mRNA target genes and target miRNAs, respectively. Fatty Acid Synthase (*FAS*), Acetyl-Coenzyme A Carboxylase alpha (*ACACA*), Fatty Acid Binding Protein 4 (*FABP4*), Peroxisome Proliferator-activated Receptor Gamma (*PPARg*), Adiponectin (*ADIPOQ*), and Retinol Binding Protein 4 (*RBP4*) were the target mRNAs. The miRNA expression levels were assessed by RT-PCR for miR-210 (*MIMAT 0000267*), miR-221 (*MIMAT 0000278*), miR-503 (*MIMAT 0002874*), miR-424 (*MIMA 0001341*), miR-378 (*MIMAT 0000732*), and miR-30c (*MIMAT 0000244*). Gene expression results are expressed, for miRNAs as well as for mRNAs, as expression ratio relative to their respective endogenous control, as described in *Materials and Methods S1*.

### Statistical Analyses

Descriptive results of continuous variables are expressed as mean ±SEM unless otherwise indicated. Before statistical analysis, normal distribution and homogeneity of the variances were evaluated using *Levene's* test. One-way ANOVA, for multiple comparisons, using post-hoc by *Bonferroni's* test when equal variances could be assumed, was used to compare groups with respect to continuous variables. Relation between quantitative variables was tested using *Pearson's t*-test. p<0.05 was considered as statistically significant. The statistical analyses and graphics were performed using the program SPSS *(Chicago, IL)* version 13.0. Microarray statistical analyses are described in detail in *Materials and Methods S1*.

## Results

### Differential miRNA Expression between Lean and Obese Cell Lines before and after *In Vitro* Differentiation

The expression of 723 human and 76 viral mature miRNAs was assessed using miRNA microarrays (*Sanger miRBase94, v10.1*) in three biological replicates of human subcutaneous fat cells (*Zen-Bio, Inc.*) from both lean (BMI<25 Kg/m^2^) and obese (BMI>30 Kg/m^2^) subjects during adipocyte differentiation (days 0, 7 and 14). Data from the replicates were very consistent.

The expression levels of 40 (5.0%) in pre-adipocytes and 31 miRNAs (3.9% of the 799 miRNAs) in mature adipocytes (14^th^ day) significantly differed between cells from lean and obese subjects (Table 1). Twenty-one of these miRNAS were common for both pre-adipocytes and adipocytes. Surprisingly, miR-923 and miR-210 levels showed an opposite expression pattern between cell lines, since both were down-regulated in obese pre-adipocytes while up-regulated in obese mature adipocytes when compared to cells from lean subjects (Table 1).

**Table 1:** Significant (p<0.0001) fold-changes for miRNA expression in *in vitro* cultured pre-adipocytes and mature adipocytes between subcutaneous fat cells from obese (BMI>30.0 Kg/m^2^) and lean (BMI<25.0 Kg/m^2^) individuals.

Groups	Pre-adipocytes (Obese vs. Lean)	Mature adipocytes (Obese vs. Lean)
*miRNAs*	*Fold-changes*	
hsa-miR-542-5p	1.61	
hsa-miR-214	1.54	
hsa-miR-181a	1.52	1.24
**hsa-miR-10a** †	**1.50**	**1.38**
hsa-let-7e	1.41	1.23
hsa-miR-503	1.39	
hsa-let-7b	1.37	1.24
hsa-miR-768-3p	1.34	1.24
hsa-miR-542-3p	1.28	
hsa-miR-30a	1.27	
hsa-miR-342-3p	1.27	1.23
hsa-miR-28-5p	1.24	
hsa-miR-410	1.24	1.21
**hsa-miR-34a** †	**1.23**	
**hsa-miR-100** †	**1.23**	
**hsa-miR-30a** ‡	**1.20**	
hsa-miR-923	*−1.21*	1.52
hsa-miR-221*	*−1.21*	
hsa-miR-34b*	*−1.21*	*−1.43*
*hsa-miR-143*	*−1.21*	
*hsa-let-7f*	*−1.22*	
*hsa-miR-193a-3p*	*−1.22*	*−1.21*
*hsa-miR-140-3p*	*−1.22*	
*hsa-miR-450a*	*−1.23*	
**hsa-miR-210** ††	***−1.24***	**1.48**
*hsa-miR-26b*	*−1.25*	
*hsa-miR-10b*	*−1.25*	*−1.25*
*hsa-miR-101*	*−1.26*	*−1.28*
*hsa-let-7c*	*−1.28*	
*hsa-miR-98*	*−1.29*	*−1.21*
*hsa-miR-23a*	*−1.30*	
hsa-miR-22*	*−1.34*	*−1.42*
*hsa-miR-337-3p*	*−1.38*	*−1.23*
*hsa-miR-31*	*−1.39*	*−1.34*
*hsa-miR-365*	*−1.43*	*−1.38*
*hsa-miR-137*	*−1.46*	*−1.45*
*hsa-miR-494*	*−1.55*	
*hsa-miR-29b*	*−1.82*	*−1.93*
***hsa-miR-221*** †	***−1.88***	***−1.84***
*hsa-miR-29c*	*−2.33*	
hsa-let-7i		1.21
***hsa-miR-125b*** †		***−1.24***
*hsa-miR-195*		*−1.22*
hsa-miR-224		1.43
*hsa-miR-26a*		*−1.26*
*hsa-miR-29a*		*−1.21*
hsa-miR-376c		1.26
*hsa-miR-424*		*−1.35*
hsa-miR-455-3p		1.48
**hsa-miR-99a** †		**1.21**

*Italic*: down-regulated in fat cells from obese.

Normal: up-regulated in fat cells from obese.

**Bold**: outstanding when integrating results from cells and subcutaneous fat tissue.

† Subcutaneous fat expression is direct and significantly (*p<0.05*) correlated with BMI.

†† Subcutaneous fat expression is inverse and significantly (*p<0.05*) correlated with BMI.

‡ Subcutaneous fat expression is down-regulated (*−1.2-fold, p = 0.03*) in Obese and DM-2 subjects.

### Study of the Changes in miRNAs Expression during Adipogenesis in Obese and Lean Cell Lines

**Figure 1:**
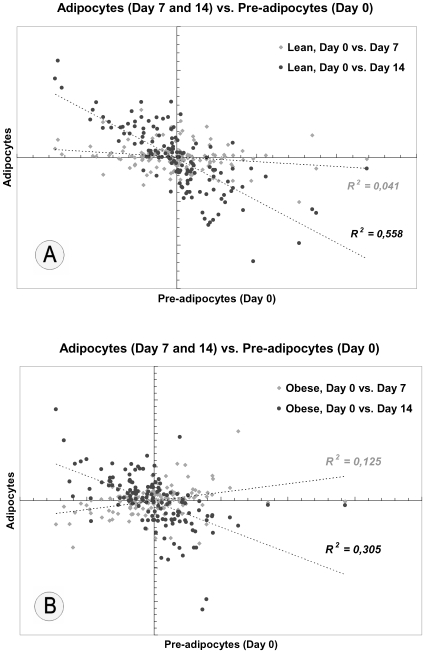
miRNA expression profiling during adipogenesis. Scatter plot showing comparison of miRNA expression profiles between undifferentiated human pre-adipocytes (Day 0) and adipocytes during differentiation at 7^th^ day (grey diamonds) or mature adipocytes at 14^th^ day (black circles) after inducing differentiation of stem-cells from subcutaneous fat depots of both lean (Fig. 1A) or obese (Fig. 1B) subjects.

After finding this differential expression in lean and obese cell lines, we aimed to study the miRNA expression profiles during adipogenesis. Interestingly, most miRNAs fell along a diagonal and negative line in the intensity scatter plot when undifferentiated cells and differentiated adipocytes were compared, indicating that the most of analyzed miRNAs were highly modulated during *in vitro* adipogenesis (Figure 1). Despite the expression profile indicated that major changes occurred in mature adipocytes (MAs) at 14^th^ day, the increased or decreased levels of the most of these miRNAs were also detected in adipocytes at 7^th^ day after starting differentiation protocol (Figure 1). The most pronounced changes for miRNAs expression pattern were detected in cells from lean subjects, in whom miRNAs expression changed more acutely between undifferentiated adipocytes and adipocytes at both day 7 and 14 (r = −0.202 and r = −0.747, respectively, p<0.0001; Figure 1A), compared with cells from the obese at day 7 and 14 (r = 0.353 and r = −0.552, respectively, p<0.0001; Figure 1B).

**Figure 2:**
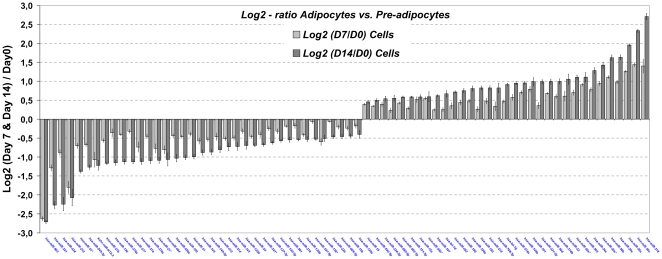
Specific miRNAs expression regulation during adipogenesis. Expressions of miRNAs significantly deregulated during differentiation. MiRNAs expression levels are normalized to an internal control and plotted as fold changes in Log2-ratio scale for day 7 vs. day 0 (grey bars) and day 14 vs. day 0 (black bars). Log2-ratios of 2-class comparisons are symmetric around 0 (while a Log2-ratio of 1 means 2-fold up-regulation, a Log2-ratio of −1 means 2-fold down-regulation as compared to day 0 miRNAs levels). Data are expressed as mean ± SEM (n = 6). All the represented miRNAs (70 of the 799 analyzed miRNAs) showed ANOVA *P*-values <0.0001 for comparisons between groups and fold-changes over 1.2-fold or under −1.2-fold day 0 miRNA expression levels.

Seventy miRNAs (8.8%) were significantly (p<0.0001) up- (37 miRNAs) or down-regulated (33 miRNAs) during adipogenesis (±1.2-fold) in subcutaneous fat cells (Figure 2). The most remarkable were miR-503 (−6.7-fold), miR-221 (−4.9-fold), miR-424 (−4.6-fold), miR-210 (−4.3-fold) and miR-31* (−2.6-fold), that were down-regulated in mature adipocytes. On the other side, miR-378 (6.6-fold), miR-30c (5.1-fold), miR-30a (4.0-fold), miR-30b (3.1-fold), miR-30e (3.1-fold), miR-30a* (2.8-fold) and miR-34a (2.5-fold), were up-regulated in mature adipocytes (Figure 2). Notably, of these 70 miRNAs, 55 were common for both obese and lean cell lines, including those that suffered the major changes.

Of those 70 miRNAs up- or down-regulated during adipocyte differentiation, 2 of the most significantly over-expressed (miR-30c and miR-378) and 4 of the most down-regulated (miR-210, miR-221, miR-424 and miR-503) were selected for validation by semi-quantitative Real Time-PCR. It should be noted that, while 3 of these miRNAs (miR-30c, miR-210 and miR-221) have been previously described as obesity and/or adipogenesis-related [**11]**, [26****], the 3 others (miR-503, miR-378 and miR-424) were not. The RT-PCR results confirmed our findings for the most of the cases in both *in vitro* and *in vivo* data [Supplemental data, SI; Figure S1].

We then analyzed the miRNA expression profile in relation with the expression of lipogenic key-factor. The expression of 6 well-known lipo/adipogenic key-genes and/or markers of adipocyte differentiation, namely Fatty Acid Synthase (FAS), Acetyl-Coenzyme A Carboxylase alpha (ACC), Fatty Acid Binding Protein 4 (FABP4), Peroxisome Proliferator-activated Receptor Gamma (PPARg), Adiponectin (ADIPOQ), and Retinol Binding Protein 4 (RBP4), was assessed by RT-PCR. As expected, the gene expression of all these factors increased during adipocyte differentiation [Figure S2, Plots]. Thus, *FAS*, *ACC*, *FABP4*, *PPARg*, *ADIPOQ* and *RBP4* gene expression levels were significantly and directly correlated with miR-30c and miR-378 but significantly and inversely related with miR-210, miR-221, miR-503 and miR-424 expression levels in RNA samples from cell lines [Figure S2].

### Study of miRNA Expression in Subcutaneous Fat from Obese Subjects

To further substantiate our findings, we studied miRNAs expression profiles in human subcutaneous adipose tissue. The expression of 799 mature miRNAs was assessed in 28 human subcutaneous fat biopsies from non-obese (n = 6) and obese with (n = 9) and without (n = 13) Type-2 Diabetes Mellitus (DM-2) women. Anthropometric and metabolic characteristics are summarized in Table S1.

**Figure 3:**
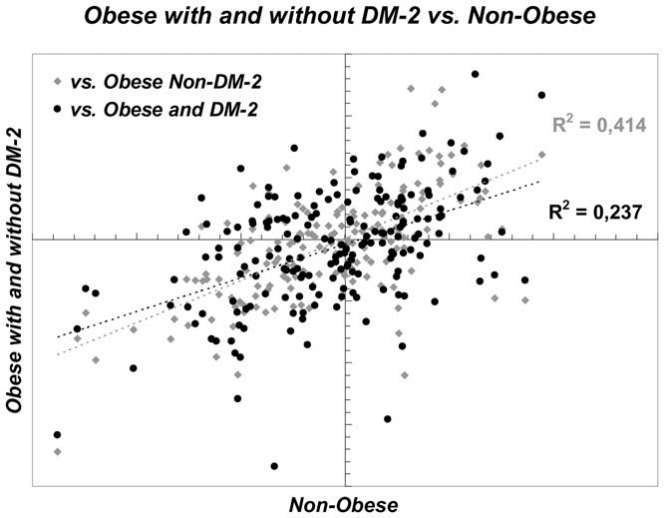
miRNAs expression profiling in human subcutaneous fat. Scatter plot showing comparison of miRNA expression profiles between subcutaneous fat from non-obese (n = 6) and obese non-DM-2 (n = 13, grey diamonds) or obese and DM-2 (n = 9, black circles) women.

**Figure 4:**
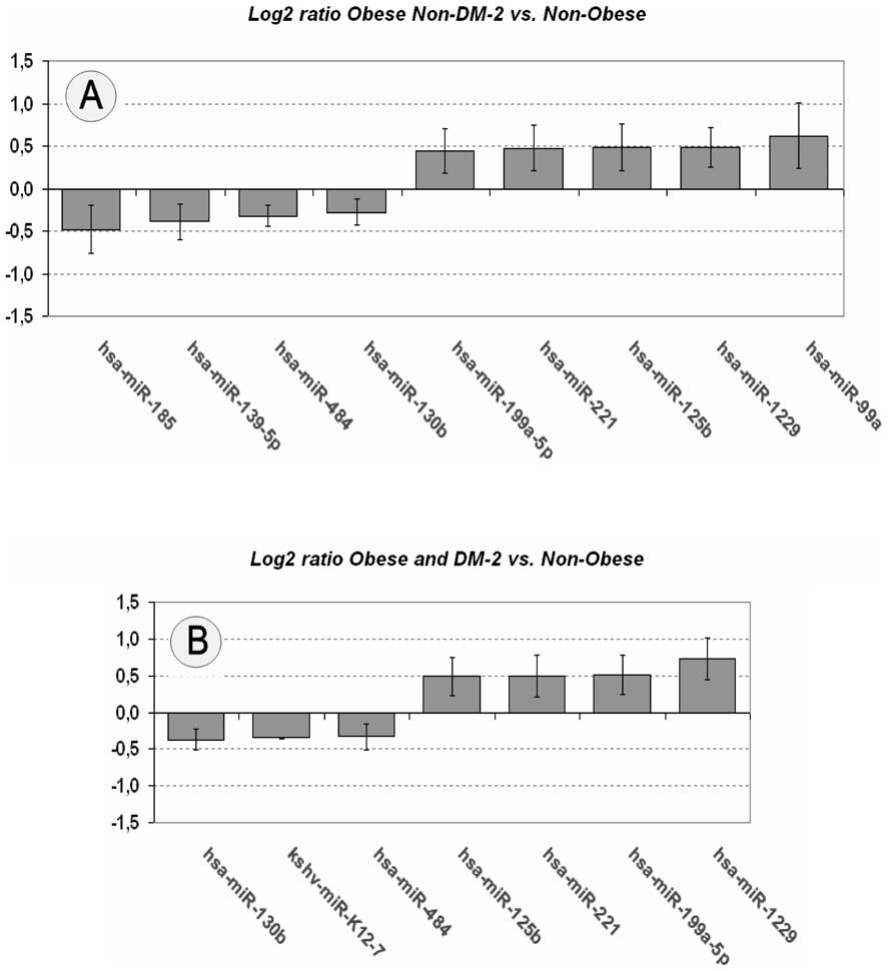
Human subcutaneous miRNAs expressions. Expressions of miRNAs are normalized to an internal control and plotted as fold-changes in a Log2-scale for obese non-DM-2 vs. non-obese (Fig. A) or obese and DM-2 vs. non-obese (Fig. B). Data are expressed as mean ± SEM. The represented miRNAs showed in each case ANOVA *P*-values <0.05 for comparisons between groups and fold-changes over 1.2-fold or under −1.2-fold subcutaneous fat from non-obese miRNA level.

Most miRNAs fell along a diagonal but positive line in the intensity scatter plot when obese (without and with DM-2) were compared to non-obese individuals (Figure 3, r = 0.64 or r = 0.49, respectively; both p<0.0001). However, the expression of 11 miRNAs (1.4% of the 799 miRNAs) significantly differed between obese with or without DM-2 and non-obese subjects [Figure 4 and Table S2]. Of note, miR-185, miR-139-5p, miR-484, and miR-130b were down-regulated in obese without DM-2 when compared to non-obese subjects while the expression of miR-99a, miR-1229, miR-125b, miR-221 and miR-199a-5p was up-regulated [Figure 4A and Table S2]. In obese subjects with DM-2, the expression of miR-K12-7, miR-484 and miR-130b, was down-regulated, while miR-1229, miR-199a-5p, miR-221 and miR-125b, were up-regulated compared with non-obese subjects [Figure 4B and Table S2]. Finally, when comparing obese subjects with DM-2 with non-DM-2 obese subjects, the only miRNA that significantly differed was miR-30a*, down-regulated (−1.2-fold, p = 0.03) in the former group [Table S2].

In concordance with our results, the expression of 17 mature miRNAs (2.1%) correlated significantly (p<0.05) with parameters of obesity (BMI) and/or lipid metabolism (fasting triglycerides) [Table S3]. Fifteen of these miRNAs were directly or inversely related to BMI. Importantly, among these 15 miRNAs, miR-130b (r = −0.406, p = 0.032), miR-210 (r = −0.362, p = 0.049), miR-100 (r = 0.411, p = 0.030), miR-221 (r = 0.436, p = 0.020) and miR-125 (r = 0.477, p = 0.010) were down-regulated during differentiation. On the other hand, miR-34a was positively associated with BMI (r = 0.385, p = 0.043) and up-regulated in mature adipocytes (Figure 2). Of note there, miR-10a, miR-34a, miR-100 (in both pre-adipocytes and mature adipocytes), miR-210 and miR-99a (only in mature adipocytes) were up-regulated in cell lines from obese subjects (Table 1) and correlated with BMI [Table S3] while miR-221 (in both pre-adipocytes and adipocytes), miR-210 (in pre-adipocytes) and miR-125b (in adipocytes) were significantly down-regulated (Table 1).

Finally, the subcutaneous fat expression of 3 miRNAs was significantly correlated with fasting triglycerides in these 28 individuals according to univariate expression analyses [Table S3]. Among miRNAs correlated with fating triglycerides, only miR-210 (r = −0.388, p = 0.040) was down-regulated during adipogenesis.

## Discussion

In this genome wide miRNA profiling study of 723 human and 76 viral in undifferentiated, *in vitro* differentiated adipocytes, and adipose tissue biopsies, the most remarkable findings were: 1) the expression of 40 (in pre-adipocytes) and 31 (in adipocytes) mature miRNAs significantly differed according to obesity; 2) the differential expression of 70 miRNAs during normal fat cell development; and 3) the expression pattern of 22 miRNAs in human subcutaneous adipose tissue was associated with parameters of adipose tissue physiology, glucose metabolism and/or obesity status. Thereby, it was possible not only to uncover the possible role of some miRNAs during normal fat cell development but also to generate a map of miRNA deregulation associated with obesity and related disorders.

### 1) Differential miRNA Expression between Lean and Obese Cell Lines before and after *In Vitro* Differentiation and during Adipogenesis

MiR-10a (in both pre-adipocytes and mature adipocytes), miR-34a, miR-100, miR-30a (only in pre-adipocytes), miR-99a and miR-210 (only in mature adipocytes) were up-regulated in cells and subcutaneous fat depots from obese when compared to those obtained from lean individuals. Otherwise, the most pronounced changes for miRNAs expression profile during differentiation were detected in cells from lean subjects.

### 2) Differential Expression of 70 miRNAs during Normal Fat Cell Development

Our genome wide miRNA screening identified, for first time, many differentially regulated miRNAs during human adipocyte differentiation. Of note were miR-221, miR-222, miR-100 and miR-125b, down-regulated during adipogenesis and associated with BMI in human adipose tissue samples. Both miR-221 and miR-222 have been described to inhibit endothelial cell migration, proliferation, and angiogenesis [**29**] while down-regulation of miR-100 and miR-125b has been associated with some malignancies [**30**]. Xie et al. also identified miR-221 and miR-125b as down-regulated during *in vitro* differentiation of mice 3T3-L1 cells [**11**]. However, miR-221 was up-regulated in adipocytes from obese mice. In the 3T3-L1 cell line, the findings with miR-100, miR-107, miR-148a and miR-30c were similar to those described here in human adipocytes [**11**].

MiR-503, the most down-regulated miRNA during differentiation, has been previously found over-expressed in retinoblastoma tumor tissue as compared to normal human retina [**31**] and involved in mouse pancreas development [**32**]. Also related to pancreas development and regulation, the cluster of miRNAs related to miR-30 (miR-30a, b, c, d and e) increased during adipocyte maturation as well as during differentiation of pancreatic islet-derived mesenchymal cells into hormone-producing cells [**32]**–[34****]. Moreover, this family of miRNAs has been recently related to cell cycle and stress response through NF-κB inactivation [**35**]. Since NF-κB has been shown as an obligatory mediator of adipose tissue inflammatory responses associated with decreased adipocyte differentiation [**36**], the observation of miR-30s up-regulation during adipocyte differentiation is in agreement with these findings.

On the other hand, miR-210 was up-regulated in pre-adipocytes and inversely associated with BMI and triglycerides. This miR-210 is linked to hypoxic stress response, a well recognized component of the tumor environment [**37**]. miR-15a, miR-101 and miR-185 were also significantly up-regulated during adipocyte differentiation in a previous work [**28**].

Our findings are also in agreement with those of Esau et al. [**27**], that identified a similar expression pattern regarding miR-30c, miR-30a*, miR-30d, miR-196, miR-107, miR-30b and miR-100 during differentiation of human adipocytes. However, these authors found that miR-143 and miR-103 were significantly up-regulated in differentiated human fat cells. We observed that miR-143 was, in fact, down-regulated during human adipocyte maturation while miR-103 did not change significantly. Similar discrepant results were observed for miR-130a and miR-130b expression levels. The origin of fat cells (fat samples from subcutaneous or visceral fat depots) could explain these discrepancies between studies.

Working with *in vitro* differentiated human adipocytes, Karbiener et al. [**38**] have recently shown the anti-adipogenic properties of miR-27b which, able to blunt the induction of PPARγ, needs to be down-regulated during adipocyte differentiation, in concordance with our *in vitro* results.

### 3) The Expression Pattern of miRNAs in Human Subcutaneous Adipose Tissue Is Associated with Parameters of Adipose Tissue Physiology, Glucose Metabolism and/or Obesity Status

Several miRNAs (miR-125b, miR-130b and miR-221) were found to be down-regulated both in mature adipocytes and in subcutaneous fat from obese subjects with or without DM-2. Klöting et al. [**26**] identified several miRNAs associated with obesity and co-morbidities. Interestingly, miR-27a, miR-140 and miR-155, down-regulated in subcutaneous fat from DM-2 patients [**26**], were also down-regulated during adipocyte differentiation. On the other hand, miR-30e expression was increased in mature adipocytes. MiR-30a* was also identified as up-regulated in mature adipocytes but down-regulated in obese and DM-2 when compared with obese non-DM-2 individuals. Klöting et al. [**26**] also described miR-210 as down-regulated in subcutaneous fat from DM-2 individuals when compared to healthy individuals. According to our data, miR-210 was down-regulated during adipocyte differentiation but not in DM-2 subjects.

Of those miRNAs significantly associated with BMI in the study by Klöting et al. [**26**], miR-34a was confirmed to be correlated with adiposity parameters, up-regulated in cultured pre-adipocytes from obese subjects and during differentiation, while miR-145 was intensely down-regulated during cell differentiation. Finally, miR-95, significantly associated with adipocyte size in the aforementioned study [**26**], increased significantly during adipocyte differentiation (current results).

The discrepant data between studies [**26**] might arise from different criteria for selecting and grouping subjects or from different technologies. In this sense, TaqMan® MicroRNA Assays technology might be more sensitive than microarrays. Moreover, it should be noted that, in the latter study, a comparative Ct method was used where miR-16 served as endogenous control to normalize the expression levels of target miRNAs. Since miR-16 seems to be, according to our data (Fig. 2), significantly increased during adipocyte differentiation, this aspect could influence significantly the reported findings [**26**].

### Integrated Results

Several miRNAs, namely miR-221, miR-125b, miR-100, miR-130b, miR-210, miR-30a*, miR-34a, miR-503 and miR-185, were outstanding when integrating results from cells and subcutaneous fat tissue together. While miR-221, miR-125b, miR-34a and miR-100 were up-regulated and miR-130b, miR-210 and miR-185 were down-regulated in obese subjects; miR-130b and miR-210 were both down-regulated during differentiation and in subcutaneous fat depots from obese subjects. However, some miRNAs were down-regulated during adipocyte differentiation and maturation (miR-221, miR-125b and miR-100) while up-regulated in obese subjects. Otherwise, miR-185 was up-regulated in mature adipocytes while down-regulated in obese men. These findings were contrary to our expectations, as far as miRNAs up-regulation both in obese subjects and in differentiated adipocytes was expected to fit, since the development of adiposity mostly depends on maturation of undifferentiated adipocytes and adipocyte hypertrophy in adults [**39]**, [40****]. This capacity of fat depots is preserved at any age from a pool of dormant precursors cells [**41**]. Only miR-34a was found to be positively up-regulated during adipogenesis and associated positively with BMI.

Current findings seem to hint at a negative relationship, with most miRNAs positively linked to adiposity and down-regulated during adipocyte differentiation. This fact could be in relation with the hyperplasic component of obesity, with increased fat cells precursors (i.e. pre-adipocytes) [**7**]. Interestingly, McLaughlin et al. [**42**] and Pasarica et al. [**43**] reported increased small adipocytes in subcutaneous fat from obese and type-2 diabetic subjects in two independent studies, suggesting the presence of an impairment in adipose cell differentiation and early maturation of pre-adipocytes linked to obesity-associated co-morbidities (such as insulin-resistance and type-2 diabetes). Of note, miR-30a*, down-regulated in obese and DM-2 when compared with obese without DM-2 subjects, was significantly up-regulated in mature adipocytes.

In summary, the remarkable inverse miRNA profile revealed for human pre-adipocytes and mature adipocytes hints at a closely crosstalk between miRNAs and adipogenesis. As far as revealed by this study, miRNAs may represent biomarkers for obesity and obesity-related complications. However, further investigations will be required to evaluate the functional consequences of these findings and the biochemical function of most of the analyzed miRNAs, which remains elusive.

## Supporting Information

Materials and Methods S1 (0.07 MB DOC)

Figure S1 Validation of microarray results. For 6 of the most regulated miRNAs in both in vitro (pre-adipocytes (▾), adipocytes (♦) at 7th day and mature adipocytes (▴) at 14th day) and in vivo (subcutaneous fat biopsies from non-obese (○), obese non-DM-2 (♦) and obese and DM-2 (□) subjects) analyses, microarray results were validated by RT-PCR assays. RT-PCR expression values for all miRNAs are the ratio relative to previously tested and validated miRNA endogenous control (RNU48). Microarray values are the ratio relative to an internal control.
(4.79 MB TIF)

Figure S2 Relationship between miRNAs and lipogenic pathway gene expression. Plots: Comparisons by RT-PCR of key-lipogenic mRNAs levels during adipogenesis. Expressions of all mRNAs are normalized to internal control (Cyclophilin A, PPIA) and plotted as fold-changes relative to this endogenous control at day 0, 7 and 14. Data are expressed as mean ± SEM (n = 6) for FAS, ACC, FABP4, ADIPOQ, PPARg and RBP4. Table: Significant correlates of miRNA and the selected adipo/lipogenic-related gene expression levels. The abbreviations used are: FAS, Fatty Acid Synthase; ACC, Acetyl-CoA Carboxilase; FABP4, Fatty Acid-Binding protein 4; PPARg, Peroxisome proliferator-activated receptor gamma; ADIPOQ, Adiponectin; RBP4, Retinol binding protein 4.
(0.25 MB EPS)

Table S1 Anthropometrical and clinical characteristics of study subjects.
(0.05 MB DOC)

Table S2 Significant fold-changes for miRNA expression in subcutaneous adipose tissue.
(0.01 MB PDF)

Table S3 Significant correlates of miRNA expression in subcutaneous fat samples.
(0.01 MB PDF)
