# Supplementary material for: MiRNA Expression Profile of Human Subcutaneous Adipose and during Adipocyte Differentiation
Source: PLoS One. 2010 Feb 2;5(2):e9022. doi: 10.1371/journal.pone.0009022 (PMC2814866; doi:10.1371/journal.pone.0009022)
Supplement: Materials and Methods S1 — (0.07 MB DOC) [file pone.0009022.s001.doc]

***SUPPLEMENTAL INFORMATION***

*Ortega et al. (November, 2009)*

***Materials and Methods S1***

***Cell culture:*** Commercially available cryopreserved human subcutaneous pre-adipocytes from two non-diabetic male subjects with age>40 and Body Mass Index (BMI) <25 or BMI>30Kg/m^2^ (SP-F-1 or SP-F-3, respectively; *Zen-Bio, Inc.*) were plated on T-75 cell culture flasks and cultured at 37ºC and 5% CO2 in Dulbecco’s modified Eagle’s medium (DMEM)/Nutrient Mix F-12 medium (1:1, v/v) supplemented with Fetal Bovine Serum (FBS) 10%, HEPES 1%, Glutamine 1% and Penicillin/Streptomycin (P/S) at 10U/mL (all from *GIBCO*, BRL; Grand Island, NY). One week later, human subcutaneous pre-adipocytes were resuspended and cultured (~40.000cells/cm^2^, 3^rd^ passage) in 6-well plates with Pre-adipocyte Medium (PM; *Zen-Bio, Inc.*) composed of DMEM/Nutrient Mix F-12 medium (1:1, v/v), FBS 10%, HEPES 1%, Glutamine 1% and P/S 1% in a humidified 37ºC incubator with 5% CO2. Twenty-four hours after plating, cells were checked for complete confluence (day 0) and differentiation was induced using Differentiation Medium (DM; *Zen-Bio, Inc.*), composed of PM with human Insulin (Ins), Dexamethasone (DXM), Isobutylmethyl-xanthine (IBMX) and PPARγ agonists (Rosiglitazone, Rs). After 7 days (day 7), DM was replaced with fresh Adipocyte Medium (AM; *Zen-Bio Inc.*), composed of DMEM/Nutrient Mix F-12 medium (1:1, v/v), FBS, HEPES, Biotin, Panthothenate, human Insulin (Ins), Dexamethasone (DXM), Penicillin, Streptomycin and Amphotericin, according to manufacturers’ guidelines. Two weeks after the initiation of differentiation (day 14), cells appeared rounded with large lipid droplets apparent in the cytoplasm. Cells were then considered mature adipocytes (MAs).

Cells from subcutaneous fat of both lean and obese individuals were harvested and stored at -80ºC at days 0, 7 and 14 after starting the differentiation protocol. Three biological replicates of fat cells from both lean and obese subjects were performed (n=3 replicates/cell/day) at days 0, 7 and 14 after starting differentiation protocol.

***Subjects and Samples******:*** Twenty-eight adipose tissue biopsies were obtained from subcutaneous fat depots during elective surgical procedures (cholecystectomy, surgery of abdominal hernia and gastric by-pass surgery), washed with cold PBS 1% P/S, fragmented and immediately flash-frozen in liquid nitrogen before be stored at -80ºC. These subcutaneous fat samples were provided from a group of 28 women with a BMI between 20 and 55Kg/m^2^. They were invited to participate at the Endocrinology Service of the *Hospital Universitari de Girona Dr. Josep Trueta* (Girona, Spain). All subjects were of Caucasian origin and reported that their body weight had been stable for at least three months before the study. They had no systemic disease other than type 2 diabetes mellitus (DM-2) and/or obesity and all were free of any infections in the previous month before the study. Liver disease and thyroid dysfunction were specifically excluded by biochemical work-up. Other exclusion criteria for those patients included the following: 1) clinically significant hepatic, neurological, or other major systemic disease, including malignancy; 2) history of drug or alcohol abuse, defined as >60g/day, or serum transaminase activity more than twice the upper limit of normal; 3) an elevated serum creatinine concentration; 4) acute major cardiovascular event in the previous 6 months; 5) acute illnesses and current evidence of high grade chronic inflammatory or infective diseases; and 6) mental illness rendering the subjects unable to understand the nature, scope, and possible consequences of the study. All subjects gave written informed consent after the purpose of the study was explained to them. The experimental protocol was approved by the Ethics Committee of the *Hospital Dr. Josep Trueta of Girona*.

***Anthropometric measurements:*** BMI was calculated as weight (in kilograms) divided by height (in meters) squared. *Deurenberg’s* formula **[1]** was used to estimate body fat composition (% fat) in all of them. According to anthropometrical and biochemical parameters such as the BMI, the % of fat mass, fasting blood glucose (ANOVA *p*-value<0.0001) [Supplemental data, SI; Table S1]**,** subjects were classified as non-obese (BMI<30.0Kg/m^2^) and obese (BMI≥30.0Kg/m^2^) with or without DM-2**.** Blood pressure was measured in the supine position on the right arm after a 10-min rest. A standard sphygmomanometer of appropriate cuff size was used and the first and fifth phases were recorded. Values used in the analysis are the average of three readings taken at 5-min intervals. Patients were requested to withhold alcohol and caffeine during at least 12h prior to the different tests. In this study, insulin resistance was measured by the homeostasis model assessment of insulin resistance (HOMA-IR) as previously reported **[2]**.

*Analytical determinations:* The serum glucose levels were measured in duplicate by the glucose oxidase method with a *Beckman Glucose Analyzer 2* *(Beckman Instruments; Brea, CA)*. The coefficient of variation (CV) was 1.9%. Serum insulin was measured in duplicate in the same centralized laboratory by monoclonal immunoradiometric assay *(Medgenix Diagnostics; Fleunes, Belgium)*. The intra-assay CV was 5.2% at a concentration of 10mU/l and 3.4% at 130mU/l. The inter-assay CVs were 6.9 and 4.5% at 14 and 89mU/l, respectively. Total serum cholesterol was measured through the reaction of cholesterol esterase/ oxidase/ peroxidase, using a *BM/Hitachi 747*. HDL cholesterol was quantified after precipitation with polyethylene glycol at room temperature. Total serum triglycerides were measured through the reaction of glycerol-phosphate-oxidase and peroxidase by routine laboratory tests on a *Hitachi 917* instrument *(Roche; Mannheim, Germany)*.

***Microarray and data analysis:*** The analysis of 799 miRNAs for both *in vitro* and *in vivo* samples was carried out in this study. Total RNA, including small RNAs and miRNAs, was extracted and purified from both adipose tissue fragments and cells using miRNeasy® Mini Kit *(QIAgen; Gaithersburg, MD)* following manufactures’ protocol. Briefly, both cells (confluent monolayer) and adipose tissue samples (~150µg) were homogenized in 0.6mL of *QIAzol® Lysis Reagent* *(QIAgen; Gaithersburg, MD)*, a monophasic solution of phenol and guanidine thiocyanate which facilitates the lysis inhibiting RNases activity. After addition of chloroform (0.4 volumes), the homogenate is separated into aqueous and organic phases by centrifugation (15min at 12,000x*g* and 4ºC). The upper aqueous phase is then extracted. For tissues with high fat content, as well as for mature adipocytes at 14^th^ day, an additional, yellow solid phase which should be avoid may be visible upper the aqueous phase. Ethanol absolute (1.5 volumes) is then added to provide appropriate binding conditions for all RNA molecules including small and micro RNAs. The sample is applied to the *RNeasy® Mini spin column* *(QIAgen; Gaithersburg, MD)*, where the total RNA binds to the membrane and phenols and other contaminants are efficiently washed away. Finally, high quality RNA is eluted in 30μL of RNase-free water.

The integrity of each RNA sample was checked with an Agilent Bioanalyzer® *(Agilent Technologies; Palo Alto, CA)*. Human miRNA microarrays *(Agilent Technologies; Palo Alto, CA)*, containing 13,737 probes corresponding to 799 miRNAs and 22 control probes, were then hybridized. Microarray hybridizations were done following the manufacture protocol with minor modifications. Briefly, 500ng of total RNA from each sample was dephosphorylated with Calf Intestinal Alkaline Phosphatase (CIP) at 37ºC for 30’ following a denaturalization and then a ligation for 2h at 16ºC using Agilent miRNA Complete Labeling and Hyb Kit *(Agilent Technologies; Palo Alto, CA)*. In this step a molecule of Cyanine3-pCp is incorporated to the 3’-end of RNA molecules. Labeled RNA was dried and resuspended with Hybridization Buffer and Blocking Agent, incubated 10min at 100ºC and transferred to an ice water bath for 5min. Samples were hybridized in a volume of 45μl to the Human miRNA V2 Oligo Microarray (Agilent) for 20 hours at 55°C and 20rpm. Microarrays were then washed at room temperature for 5min in Gene Expression Wash Buffer 1 and 5min at 37ºC in Gene Expression Wash Buffer 2 *(Agilent Technologies; Palo Alto, CA)*.

Microarrays with miRNA content corresponding to miRbase v10.1 (six probes for each miRNA on one chip) were used for profiling human pre-adipocytes, mature adipocytes and subcutaneous fat biopsies from non-obese and obese with or without DM-2 subjects. Arrays were scanned on an Agilent G2565BA microarray scanner under default settings recommended by Agilent Technologies for miRNA microarrays with 100% PMT and 5μm resolution. Data was extracted using Agilent Feature Extraction Software *(Agilent Technologies; Palo Alto, CA)*. Extracted Log2-transformed intensities were quartile normalized to make all data comparable. Microarray probes were collapsed to miRNAs by taking the median expression values of the respective probes per miRNA. Differential expression was analyzed using Significance Analysis of Microarrays (SAM) applied to the ionizing radiation response **[3]** for the tissue samples and time course analysis **[4]** for the cell lines. We corrected for multiple testing using the False Discovery Rate (FDR) method **[5]**. All statistical analyses were performed with the Bioconductor project (v2.3) in the *R* statistical environment (v2.8.1) **[6]**. Within the miRNAs with significant test results, we report only those which have normalized (non-logged) signal intensities above 150 in at least half of the samples involved in the respective comparison, and which hence can be trusted not to represent "*noise*".

Complete miRNA microarray data for cells and fat depots were deposited into Gene Expression Omnibus (GEO) following MIAME compliant guidelines (Accession numbers: GSE18469 and GSE18470, respectively; NCBI tracking system #15713167). MiRNAs described in this paper are referred to their names in miRbase v10.1.

*Gene expression analyses:* Three μg of total RNA were reverse transcribed to cDNA using High Capacity cDNA® Archive Kit *(Applied Biosystems; Darmstadt, Germany)* according to the manufacturers’ protocol. MiRNAs were specify reverse transcribed by TaqMan® MicroRNA Reverse Transcription Kit *(Applied Biosystems; Darmstadt, Germany)* which provides the necessary components for optimal performance of TaqMan® MicroRNA Assays by Real Time-PCR (RT-PCR). Gene expression was then assessed by RT-PCR using an ABI Prism® 7000 Sequence Detection System *(Applied Biosystems; Darmstadt, Germany)* and TaqMan® technology suitable for relative gene expression quantification. The reaction was performed for mRNAs as well as for miRNAs following manufacturers’ protocol in a final volume of 25μl. The cycle program consisted of an initial denaturing of 10min at 95ºC then 40 cycles of 15sec denaturizing phase at 92ºC and 1min annealing and extension phase at 60ºC. Positive and negative controls were included in all the reactions.

The commercially available and pre-validated TaqMan® primer/probe sets used were as follows: Cyclophilin A (*PPIA; Hs99999904_m1, RefSeq. NM_ 002046.3*) was used such as endogenous control for mRNA target genes in each reaction and Fatty Acid Synthase (*FAS; Hs00188012_m1, Ref.Seq. NM_ 004104.4*), Acetyl-Coenzyme A Carboxylase alpha (*ACACA; Hs00167385_m1, Ref.Seqs. NM_ 198834.1, NM_ 198836.1, NM_ 19883 7.1, NM_ 198838.1 and NM_ 198839.1*), Fatty Acid Binding Protein 4 (*FABP4 or aP2; Hs00609791_m1, Ref.Seq. NM_ 001442.2*), Peroxisome Proliferator-activated Receptor Gamma *(PPARg; Hs01115513_m1, Ref.Seqs. NM_ 138711.3, NM_ 138712.3, NM_ 005037.5 and NM_ 015869.4)*, Adiponectin *(ADIPOQ; Hs00605917_m1, Ref.Seq. NM_ 004797.2)*, and Retinol Binding Protein 4 *(RBP4; Hs00924047_m1, Ref.Seq. NM_ 006744.3)* were the target mRNAs.

To normalize miRNAs expression by RT-PCR two endogenous controls were tested (RNU48 and RNU6B), following manufacturers’ guidelines. Since RNU48 displayed lower variability than RNU6B across both cells (Standard Deviation of the average threshold cycle (Ct) of 0.4 vs. 0.6, respectively) and fat samples (0.5 vs. 0.6, respectively), RNU48 was finally selected as the best internal control for normalizing miRNAs RT-PCR results from both *in vivo* and *in vitro* samples. The miRNA expression levels were assessed by RT-PCR for the miR-210 (MIMAT 0000267), miR-221 (MIMAT 0000278), miR-503 (MIMAT 0002874), miR-424 (MIMA 0001341), miR-378 (MIMAT 0000732), and miR-30c (MIMAT 0000244) by TaqMan® MicroRNA Assays.

Data were then analyzed by the relative quantification method (2**^-∆Ct^**). A threshold cycle (Ct value) was obtained for each amplification curve and a ∆Ct value was first calculated by subtracting the Ct value for the appropriate endogenous control from the Ct value for each sample and transcript. Fold changes compared with the endogenous control were determined by calculating 2**^-∆Ct^**, so gene expression results are expressed for miRNAs as well as for mRNAs as expression ratio relative to the endogenous control according to manufacturers’ instructions.

***References***

1. Deurenberg P, van der Kooy K, Leenen R, Weststrate JA, Seidell JC (1991) Sex and age specific prediction formulas for estimating body composition from bioelectrical impedance: a cross-validation study. Int J Obes 15: 17-25.

2. Matthews DR, Hosker JP, Rudenski AS, Naylor BA, Treacher DF, et al. (1985) Homeostasis model assessment: insulin resistance and beta-cell function from fasting plasma glucose and insulin concentrations in man. Diabetologia 28: 412-419.

3. Tusher VG, Tibshirani R, Chu G (2001) Significance analysis of microarrays applied to the ionizing radiation response. Proc Natl Acad Sci U S A 98: 5116-5121.

4. Conesa A, Nueda MJ, Ferrer A, Talon M (2006) maSigPro: a method to identify significantly differential expression profiles in time-course microarray experiments. Bioinformatics 22: 1096-1102.

5. Benjamini Y, Drai D, Elmer G, Kafkafi N, Golani I (2001) Controlling the false discovery rate in behavior genetics research. Behav Brain Res 125: 279-284.

6. Gentleman RC, Carey VJ, Bates DM, Bolstad B, Dettling M, et al. (2004) Bioconductor: open software development for computational biology and bioinformatics. Genome Biol 5: R80.
