# Supplementary figures and images for: MiRNA Expression Profile of Human Subcutaneous Adipose and during Adipocyte Differentiation
Source: PLoS One. 2010 Feb 2;5(2):e9022. doi: 10.1371/journal.pone.0009022 (PMC2814866; doi:10.1371/journal.pone.0009022)

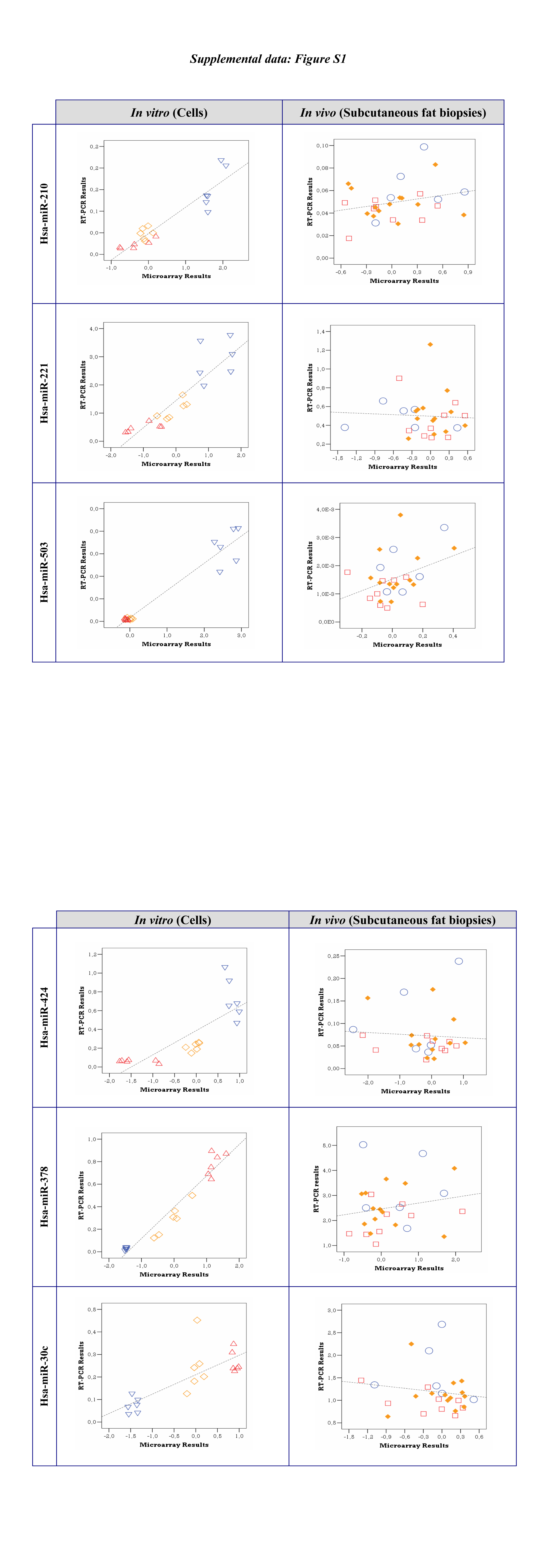

Supplement: Figure S1 — Validation of microarray results. For 6 of the most regulated miRNAs in both in vitro (pre-adipocytes (▾), adipocytes (♦) at 7th day and mature adipocytes (▴) at 14th day) and in vivo (subcutaneous fat biopsies from non-obese (○), obese non-DM-2 (♦) and obese and DM-2 (□) subjects) analyses, microarray results were validated by RT-PCR assays. RT-PCR expression values for all miRNAs are the ratio relative to previously tested and validated miRNA endogenous control (RNU48). Microarray values are the ratio relative to an internal control. (4.79 MB TIF) [file pone.0009022.s002.tif]
