# Supplementary material for: MiRNA Expression Profile of Human Subcutaneous Adipose and during Adipocyte Differentiation
Source: PLoS One. 2010 Feb 2;5(2):e9022. doi: 10.1371/journal.pone.0009022 (PMC2814866; doi:10.1371/journal.pone.0009022)
Supplement: Table S1 — Anthropometrical and clinical characteristics of study subjects. (0.05 MB DOC) [file pone.0009022.s004.doc]

***Table S1*** *Anthropometrical and clinical characteristics of study subjects.* The abbreviations used are: BMI, Body mass index; % fat, percent body fat-mass estimated by *Deurenberg’s* formula; hA1c, Glycosylated hemoglobin; HOMA-IR, Homeostasis model assessments of insulin-resistance; DM-2, Type-2 Diabetes Mellitus.

|  | *Non-Obese* | *Obese without DM-2* | *Obese with DM-2* | *p-* value |
| --- | --- | --- | --- | --- |
| ***n*** | 6 | 13 | 9 |  |
| ***Age (years)*** | 42 ±9 | 41 ±9 | 45 ±10 | 0.622 |
| ***BMI (kg/m^2^)*** | **26.6 ±2.7** | **44.5 ±3.0** | **44.5 ±4.9** | ***<0.0001*** |
| ***% fat*** | **36.4 ±4.8** | **56.3 ±4.8** | **58.0 ±6.4** | ***<0.0001*** |
| ***Blood glucose (mg/dl)*** | **85.0 ±12.1** | **92.9 ±13.7** | **139.4 ±25.7** | ***<0.0001*** |
| ***Fasting insulin (μIU/mL)*** | 17.0 ±14.3 | 13.4 ±7.8 | 13.9 ±5.0 | 0.857 |
| ***HOMA-IR*** | 1.4 ±1.2 | 3.3 ±1.9 | 3.7 ±1.8 | 0.613 |
| ***HbA_1c_*** | 4.4 ±0.2 | 4.9 ±0.4 | 5.7 ±1.4 | 0.050 |
| ***Total cholesterol (mg/dl)*** | 197.3 ±37.9 | 193.1 ±27.4 | 206.9 ±30.4 | 0.576 |
| ***HDL-cholesterol (mg/dl)*** | **84.5 ±33.8** | **62.7 ±11.2** | **51.3 ±7.6** | ***0.012*** |
| ***LDL-cholesterol (mg/dl)*** | 91.3 ±49.9 | 113.1 ±19.4 | 127.2 ±26.5 | 0.141 |
| ***Fasting Triglycerides (mg/dl)*** | 115.2 ±37.4 | 99.1 ±41.9 | 120.5 ±39.6 | 0.467 |
| ***Systolic blood pressure (mmHg)*** | 115.8 ±12.7 | 134.6 ±14.5 | 140.8 ±18.7 | 0.053 |
| ***Diastolic blood pressure (mmHg)*** | 68.3 ±8.5 | 76.7 ±9.7 | 81.0 ±13.1 | 0.233 |
