# Supplementary material for: MiRNA Expression Profile of Human Subcutaneous Adipose and during Adipocyte Differentiation
Source: PLoS One. 2010 Feb 2;5(2):e9022. doi: 10.1371/journal.pone.0009022 (PMC2814866; doi:10.1371/journal.pone.0009022)
Supplement: Table S2 — Significant fold-changes for miRNA expression in subcutaneous adipose tissue. (0.01 MB PDF) [file pone.0009022.s005.pdf]

Table S2 Significant (p<0.05) fold-changes for miRNA expression in subcutaneous fat samples from obese with or without DM-2 patients and healthy and lean individuals.

**Groups**	**Obese Non-DM-2 vs. Non-Obese**	**Obese and DM-2 vs. Non-Obese**	**Obese and DM-2 vs. Obese Non-DM-2**
miRNAs		*Fold-changes (P-value)*	
**hsa-miR-99a**	1.5-fold (p=0.04)		
**hsa-miR-1229**	1.4-fold (p=0.04)	1.7-fold (p<0.01)	
**hsa-miR-125b**	1.4-fold (p=0.02)	1.4-fold (p=0.02)	
**hsa-miR-221**	1.4-fold (p=0.02)	1.4-fold (p=0.02)	
**hsa-miR-199a-5p**	1.4-fold (p=0.02)	1.4-fold (p=0.01)	
**hsa-miR-130b**	-1.2-fold (p=0.04)	-1.3-fold (p=0.02)	
**hsa-miR-484**	-1.3-fold (p=0.01)	-1.3-fold (p=0.02)	
**hsa-miR-139-5p**	-1.3-fold (p=0.03)		
**hsa-miR-185**	-1.4-fold (p=0.04)		
**kshv-miR-K12-7**		-1.3-fold (p=0.04)	
**has-miR-30a***			-1.2-fold (p=0.03)
