# Supplementary material for: MiRNA Expression Profile of Human Subcutaneous Adipose and during Adipocyte Differentiation
Source: PLoS One. 2010 Feb 2;5(2):e9022. doi: 10.1371/journal.pone.0009022 (PMC2814866; doi:10.1371/journal.pone.0009022)
Supplement: Table S3 — Significant correlates of miRNA expression in subcutaneous fat samples. (0.01 MB PDF) [file pone.0009022.s006.pdf]

Table S3 Significant correlates of miRNA expression in subcutaneous fat samples.

	BMI	***Fasting Triglycerides***
	r (p-value)	***r (p-value)***
**hsa-miR-99a**	0.502 (p=0.006)	
**hsa-miR-125b**	0.477 (p=0.010)	
**hsa-miR-221**	0.436 (p=0.020)	
**hsa-miR-199b-3p**	0.434 (p=0.021)	
**hsa-miR-199b-5p**	0.412 (p=0.030)	
**hsa-miR-100**	0.411 (p=0.030)	
**hsa-miR-10a**	0.409 (p=0.031)	
**hsa-miR-146a**		0.407 (p=0.039)
**hsa-miR-34a**	0.385 (p=0.043)	
**hsa-miR-199a-5p**	0.384 (p=0.044)	
**hsa-miR-1229**	0.380 (p=0.046)	
**hsa-miR-210**	-0.362 (p=0.049)	-0.388 (p=0.040)
**hsa-miR-99b**		-0.392 (p=0.048)
**hsa-miR-92a**	-0.398 (p=0.036)	
**hsa-miR-142-5p**	-0.402 (p=0.034)	
**hsa-miR-130b**	-0.406 (p=0.032)	
**hsa-miR-484**	-0.510 (p=0.006)	
